# Stages of Biological Development across Age: An Analysis of Canadian Health Measure Survey 2007–2011

**DOI:** 10.3389/fpubh.2017.00355

**Published:** 2018-01-11

**Authors:** Yi-Sheng Chao, Hsing-Chien Wu, Chao-Jung Wu, Wei-Chih Chen

**Affiliations:** ^1^Centre de recherche du centre hospitalier de l’Université de Montréal (CRCHUM), Université de Montréal, Montréal, QC, Canada; ^2^Taipei Hospital, Ministry of Health and Welfare, New Taipei City, Taiwan; ^3^Département d’informatique, Université du Québec à Montréal, Montréal, QC, Canada; ^4^Department of Chest Medicine Taipei Veterans General Hospital, Taipei, Taiwan; ^5^Faculty of Medicine, School of Medicine, National Yang-Ming University, Taipei, Taiwan

**Keywords:** life stages, principal components, principal component analysis, Canadian Health Measures Survey, biomarkers

## Abstract

**Introduction:**

The stages of biological development are not clearly defined despite the fact that they have been used to refer to concepts such as adolescence and aging. This study aimed to (1) propose and test a framework to search for stages of representative components and determine stages of stability and transition, (2) identify stages of biological development based on health questionnaire and biomarker data, and (3) interpret the major trajectories in a health and biomarker database.

**Methods:**

This study analyzed the data on the Canadian Health Measures Survey (CHMS) interviewees from cycle 1 to 3 (2007–2013) in Canada. We selected 282 variables containing information from questionnaire and on biomarkers after removing redundant variables based on high correlation. Fifty-nine nominal variables were replaced by 122 binominal variables, leaving 345 variables for analysis. Principal component (PC) analysis was conducted to summarize the data and the loadings were used to interpret the PCs. A stable stage was assumed to be the age groups without significantly different values of PCs.

**Results:**

The CHMS interviewed 16,340 Canadians. Of all, 51.25% were female. The age ranged from 6 to 79 years (mean = 34.41 years, 95% CI = 34.74–34.08). The proportions of total variance explained by the first three PCs were 12.14, 4.03, and 3.19%, respectively. The differences of the first PC were not significant, especially between age 22 and 33, 34 and 40, 41 and 45, 46 and 71, and 72 and 79 years (adjusted *p* > 0.05 for all). The leading variable, in terms of the variance contributed to PC1, was time spent in physical activities, followed by variables related to alcohol consumption, and smoking. The 13 leading contributors to PC2 variances were all lung function measures.

**Discussion and conclusion:**

There are stages of stability and transition across all age groups based on the first PCs. The first and second PCs are related to physical development and lung function. The identification of stable stages is the first step to understanding how human biology develops in a population perspective and will be important for research that relies on a research population with similar characteristics to draw samples for observation or intervention.

## Introduction

There is a variety of biological and physical measures, which professionals have proposed to describe the process of aging and human development. For example, the changes in pulmonary function have been useful in identifying peak ages of respiratory capacity and unique disease patterns, such as respiratory restriction and obstruction (1). Another example is that cognition deterioration has been emphasized as an aging characteristic in later life (2, 3). However, the complex nature of human biology has prevented researchers from summarizing overall trajectories to better classify stages of human development and aging (4). As a consequence, these stages, such as adolescence and old age, are frequently referred to without explicit definition or clarification (5).

Identifying the stages within a life course is also the key to answering important questions, such as when the process of aging begins (6) and what characteristics can be used to define healthy aging and developmental trajectories. The identified stages will also be useful for researchers who prefer a life course perspective that aims to link exposure in early life to outcomes in advanced age (5). Currently, there are theories on the trajectories of life (7) to show that health trajectories diverge at the end of life (8–10) or physiological functions evolve with different developmental stages (2, 11). The evidence on important issues, such as the beginning of aging (6) and the definitions of life stages (12), remains scarce. We find it necessary to introduce population data and propose a framework to define the representative stages across the lifespan (13).

One of the major biobank projects, the Canadian Health Measures Survey (CHMS), is a prominent example of exhaustive efforts to document biology of human development and aging at a population level (14–16). It documents not only the biological measures that need to be quantified with various instruments, such as lung functions and blood chemistry, but also factors related to human physiology, such as diet, smoking, physical activities, and life style (16). The CHMS is also important for its capacity of producing nationally representative statistics and a wide age range from 6 to 79 years. The focus on the biomarkers and the concurrent collection of other covariates make the CHMS an optimal source of information.

There are three objectives in this study. First, we aim to propose and test a framework to search for stages of representative components and determine stages of stability and transition accordingly, taking principal components (PCs) derived from principal component analysis (PCA) for example. We assume that the stages of representative components should consist of consecutive age groups and the values within a stable stage should not be significantly different. Second, we aim to identify stages of biological development based on health questionnaire and biomarker data. Finally, we aim to interpret the major trajectories according to the leading contributors to representative components.

## Materials and Methods

### Data Sets

This study analyzed the three cycles of the cross-sectional CHMS that aimed to investigate biomarkers and related measures since 2007 (15, 17). The first, second, and third cycles were conducted from 2007 to 2009, from 2009 to 2011, and from 2012 to 2013, respectively (18). In each cycle, it interviewed more than 5,000 participants aged 3 (cycle 2 and 3) or 6 (cycle 1) to 79 years residing in the following Canadian provinces: Ontario, Quebec, Atlantic provinces, Prairie provinces, and British Columbia (16). The CHMS had the sampling frame that covered more than 95% of Canadians (16). The population excluded from being sampled were people living in the three territories, people living on reserves or aboriginal settlements, institutionalized individuals, full-time members in the Canadian Forces (16). There were household and clinic questionnaires administered. The CHMS interviewed participants for life style, socioeconomic status, residence status, diets, medical history, and family history (16). There were mobile clinics that collected biological specimens or took physiological measurements (16). There were direct physical and biological measures in the following dimensions common to three cycles: anthropometry, spirometry, blood pressure, fitness, physical activity, oral health examination, and blood and urine specimens (17). These components were assumed to be important for staging representative components and were included for analysis.

### Data Linkage and Processing

**Figure 1:**
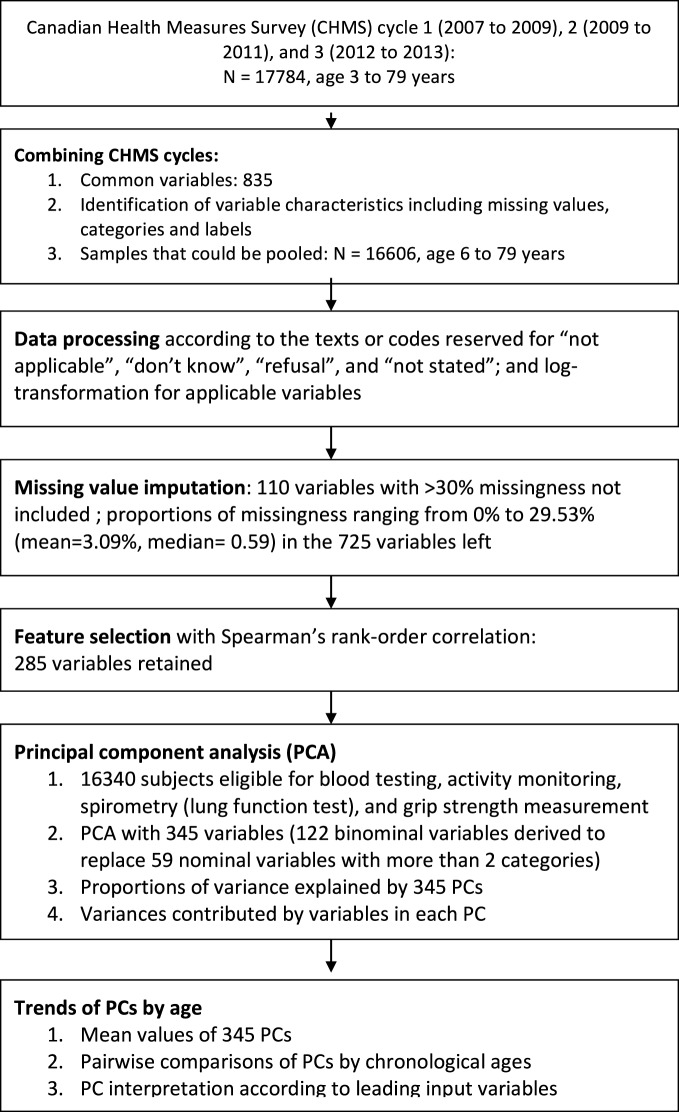
Flow chart of data linkage, data processing, and feature selection with the Canadian Health Measures Survey (CHMS) cycle 1 to 3.

The three cycles of the CHMS data were pooled and merged by 835 common variables names (see Supplementary Material for the list of variables and their characteristics; see Figure 1 for the flowchart of this study). Administrative variables were identified and variables that were used to derive final summary measures were not used for analysis. For example, the frequencies of leisure activities were reported in times per day, week, and year to produce the annual summaries. Only the annual frequencies were retained for analysis.

#### Data Pre-Processing and Recoding

The variables measured by different categorizations across three cycles were recoded for consistency, including measures in the frequencies or numbers of computer use, video games, watching television, and reading (variable names: sac_11, sac_13, and sac_14). These variables were measured in continuous scales in cycles 2 and 3, but measured in categories in cycle 1. The variables representing ages of disease onset were replaced with years since disease occurrence. The “not applicable” category in the variables that designated for years since disease occurrence or numbers of activities were replaced with 0, while the same categories in other variables were retained (see Supplementary Material for the proportions of missing values or “not applicable” in the variables).

The codes or texts represented “don’t know,” “refusal,” or “not stated” were recoded to “no data” in each variable (see Supplementary Material for the proportions of missingness). The variables that had more than 30% of unweighted subjects with missing values were not included for analysis. For this reason, the variables representing the fifth to eighth measures of lung function tests were not included for analysis (maximum eight trials documented). The missing values were then imputed with multiple imputation chained equations (*mice* package available from R package repository) (19). The biomarker variables without any missing values were used to impute missing values in other variables (see Supplementary Material for the biomarkers without missing values).

#### Feature Selection with Correlation

We first selected variables with a correlation-based method that was designed in part to remove redundant variables and increasing computational feasibility (20, 21). The data redundancy might be created for the ease of survey implementation, data processing, or concerns on measurement failures. For example, the food frequencies were reported in daily, weekly, and annually intake before the annual summaries were obtained. However, only the annual frequencies were used for analysis. Blood pressure was measured for multiple times and the mean values of adequate measures were derived. Up to eight trials of lung function tests were documented.

A correlation matrix of all variables, categorical or continuous, was created (20, 21). Because the missing values were imputed, there were no subjects excluded from correlation analysis. We used coefficients greater than 0.9 as the threshold to exclude redundant variables (22). There were 285 variables left for further analysis (see Figure 1 for the flowchart; see Supplementary Material for the list of variables used for PCA).

#### Variable Transformation

The skewness of all continuous variables was evaluated and log transformation was applied for the variables whose skewness could be reduced after transformation. Ordinal variables that had answers in an order that represented severity or relative scales were retained in original values, including numbers of residents of specific ages, general health status, and alcohol consumption frequencies (see Supplementary Material). There were 59 nominal variables with more than two categories, which did not represent specific orders, and were transformed and replaced by 122 binominal variables. This led to a total of 345 variables for further analysis.

#### Principal Component Analysis

Principal component analysis had been used for dimension reduction and data pre-processing (23). There was a variety of PCA methods (23–25) and related data summary methods (26, 27), such as regularized PCA, kernel PCA, and multicenter PCA. For simplicity and the survey design in the CHMS, linear PCA was deemed optimal and first tried to test the concept of staging representative components in the CHMS data sets (28). Prior to PCA, each variable was centered to 0 and scaled to unit variance (26, 27). The differences in PC values between any two age groups were tested with *t*-tests to obtain statistical significances.

The contributions of variable variance to each PC were estimated in two steps (13). First, the loading coefficient of each variable in each PC was retrieved and then squared, ranging from 0 to 1 due to unit variance. The PC values were interpreted according to the PCA loadings (13, 29). The contribution of the *i*-th input variables was then defined as the product of the PC variances and the *i*-th squared values for each PC, *i* denoting the order of the input variables based on the absolute values of the PCA loadings.

#### Definition of Stages of Biological Development

The representative components for staging were the leading PCs selected according to the screen test (30). We group the subjects by age, from 6 to 79 years old. Each age group consisted of subjects of the same age, and totaled 74 groups. *p*-Values less than 0.05 were considered statistically significant and were adjusted for multiple comparison with the Benjamini–Hochberg method (31). A group of consecutive age subsets was defined as stable if less then 5% of all pairs of age subsets were significantly different. The stages of representative components were searched in the following manner. We began with age 0 and examined whether the PC value at the age group was significantly different from the age 1. Without significant difference in PC values, a *stable stage* consisting of age 0 and 1 was identified. We continued the process and assumed that we recognized a stage consisting of age 0 to age *i* subsets, *i* being greater than one. We then determined whether the stage which included age 0 and age *i* + 1 subsets was a stable stage. We stopped the search if age *j, j* greater than *i*, did not belong to the stable stage consisting of age 0 to *j* − 1. We restarted the process from the age *j* subset and considered the stable stage consisting age 0 to *j* − 1 as a separate group. This process was repeated until the 79-year age subset. The ages between any two stable stages were considered stages of transition. This study adopted R (32) (v. 3.24 released in March 2016) and R Studio (33) for data analysis.

## Results

There were 16,606 subjects available in cycle 1 to 3 of the CHMS between 2007 and 2013. Of all, 16,340 were eligible for blood testing, activity monitoring, spirometry (lung function test), and grip strength measurement (see Figure 1 for the flowchart). In Table 1, the numbers of subjects and the demographic characteristics were tabulated by survey cycles. The proportions of males and females were 48.75 and 51.25%. The mean age was 34.41 years (95% CI = 34.74–34.08).

**Table 1:** Demographic characteristics of the populations in the Canadian Health Measures Surveys from cycle 1 to 3, 2007 to 2013.

Cycles	Years	Sample sizes (*n*)	Female (%)	Age (years)	95% CI
1	2007–2009	5,528	51.68	34.64	35.21–34.06
2	2009–2011	5,673	51.95	34.33	34.89–33.78
	2012–2013	5,139	50.03	34.25	34.85–33.65
All		16,340	51.25	34.41	34.74–34.08

### Variables Contributing to the Variability of First PCs

For the first objective, we found that PCA was feasible and the PCs were useful for staging. The proportions of total variance explained by the first five PCs were 12.14, 4.03, 3.19, 2.04, and 1.72%, respectively. Each of the first 11 PCs explained more than 1% of total variance.

### Stable Stages of PCs

**Figure 2:**
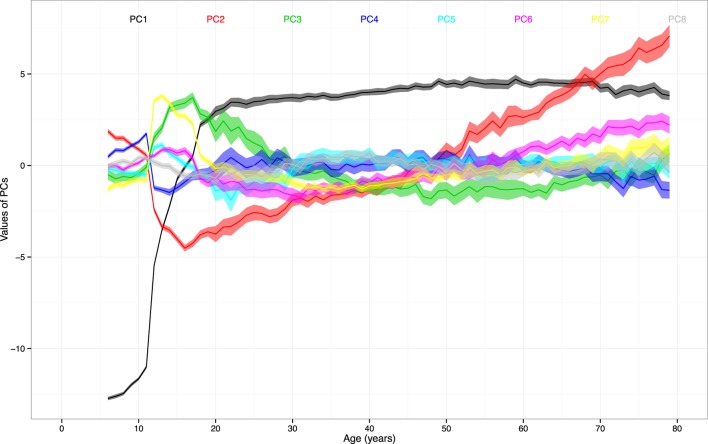
The mean values and 95% CIs of the first to eighth principal components (PCs) by age. Note: ranges around colored lines = 95% confidence intervals (CIs); colors and corresponding numbers labeled on the graph.

For the second objective, we identified the stages of stability and transition based on the PCs. The mean values of the first to sixteenth PCs were plotted in Figure 2 to highlight the larger magnitude of changes in the PC1 and PC2. The first eight PCs had changes of greater magnitudes than the rest of PCs across the age range. There was a sharp incline of PC1 values between ages 10 and 20. The values of PC2 declined until age 16 and inclined thereafter.

By leaving out the insignificant differences, there seemed to be cutoff ages that separate certain stages of stability and transition. The differences of PC1 were not significant from age 22 to 33, 34 to 40, 41 to 45, 46 to 71, and 72 to 79 (adjusted *p* values > 0.05 for all). The differences of PC2 were not significant from age 18 to 22, 23 to 28, 31 to 37, 38 to 41, 45 to 48, 49 to 52, 56 to 60, 68 to 71, and 75 to 78 (adjusted *p*-values > 0.05 for all).

### Interpretation of the PCs

For the last objective, we found that there were patterns observed in the PCA loadings that can be used to interpret PCs. In Table 2, the 40 leading variables were sorted by the contributed variances for PC1 (first PC). The leading variable, in terms of the variance contributed to PC1, was time spent in physical activities, followed by variables related to alcohol consumption, smoking, grip strength, respiratory functions or symptoms, height and weight, blood markers (alkaline phosphatase, anti-hepatitis B core antigen, and phosphate), and self-perceived mental health. There were four variables contributing more than 70% of own variable variance to PC1. The PC1 might be interpreted as a component consisting of variables representative of physical activities and life style.

**Table 2:** The contribution of variance of each variable to PCs, sorted by the values in the first five PCs.

Variables	Labels	PC1	PC2	PC3	PC4	PC5
cpa_14	Hours/week—physical activity in class	0.79	0.024	0.005	0.038	0.006
alcdtyp	Type of drinker—(d)	0.744	0.008	0.006	0	0.003
gsmd53	Grip strength norms for respondents <15 years old (d)	0.719	0	0.003	0.006	0
lab_hbc.negative	Anti-HBC	0.705	0.007	0	0.007	0
bpmdbpk	Blood pressure norms for respondents less than 18 (d)	0.697	0.033	0.042	0.001	0
spq_23	Simple chores make short of breath	0.695	0.002	0.006	0.01	0
spq_25	Frequent persistent colds	0.692	0.006	0.005	0.019	0.001
spq_22	Cough up phlegm regularly	0.68	0	0.003	0.003	0
sdq_29	Picked on or bullied by other children/youth	0.676	0.03	0.036	0.001	0
hwtdcol	BMI class (12–17)/self-reported—(d)	0.67	0.037	0.043	0	0
hwtdhtm	Height (m)/self-reported—(d)	0.662	0.085	0.004	0.029	0.001
hwtdlb	Weight (pounds)/self-reported—(d)	0.652	0.001	0.002	0.06	0.004
lab_alkp	Alkaline phosphatase (U/l)	0.633	0.001	0.005	0.021	0
smk_60.no	Smoke cigar/pipe; used snuff or chew tobacco-past month	0.62	0.007	0.002	0.078	0.015
gen_15	Self-perceived stress	0.609	0	0	0.014	0.001
cpa_17	Hours/day—watching TV	0.607	0.019	0.004	0.034	0.01
ccc_82.no	Has a thyroid condition	0.599	0.068	0.007	0.017	0.002
spq_24.no	Wheeze from exertion or at night	0.573	0.047	0	0.052	0.012
gen_14	Self-perceived mental health	0.563	0.043	0	0.036	0.016
bpmdbpa	Blood pressure norms for respondents 18 or older (d)	0.558	0.003	0.028	0.004	0.001
sxb_21.no	Ever diagnosed with a sexually transmitted disease	0.551	0.019	0.022	0.001	0.002
hwmdlln	Leg length in centimeters (d)	0.541	0.097	0.005	0.039	0.002
gen_19	Self-perceived quality of life	0.511	0.052	0	0.048	0.018
lab_phos	Phosphate (mmol/l)	0.506	0	0.009	0	0.002
smk_24	# cigarettes/d smoked @ age at least 1 cig/month	0.502	0.052	0.006	0.075	0.015
ccc_45.no	Has COPD	0.479	0.107	0.102	0.001	0.01
gsm_12	Left hand grip strength (1)	0.476	0.131	0.001	0.156	0
gsmd52	Grip strength norms respondents <15 years old (d)	0.463	0.022	0.001	0.019	0
bri_12	Length of time breastfed	0.463	0.016	0.003	0.028	0.004
spq_21.no	Cough regularly	0.463	0.041	0	0.064	0.018
gen_18	Sense of belonging—local community	0.443	0.015	0.001	0.047	0.019
edudr10	Highest level of education—respondents. 10 levels—(d)	0.443	0.002	0.051	0.02	0
bri_13	Length of time fed only breast milk	0.434	0.014	0.003	0.025	0.005
dhh_ms.single	Marital status	0.433	0.089	0.086	0	0.023
spm_pv1f	Respondent’s predicted FEV1/FVC (%)	0.431	0.213	0.065	0.012	0
smk_12	Type of smoker	0.424	0.001	0.017	0.016	0.022
hwtdbmia	BMI class adults 18 and older—international standard	0.42	0.001	0.05	0.017	0.01
spm_pv1	Respondent’s predicted FEV1 (l)	0.41	0.335	0.015	0.103	0.005
hwmdbmi	Body mass index—(d)	0.405	0.04	0.002	0.015	0.017
slp_11	Number hours spent sleeping per 24 h	0.4	0.001	0.005	0.001	0

*The numbers or texts after the “.” in the variable names indicate the categories of nominal variables. Other variables are not shown for limited space. The maximum variance of each variable to contribute is one. See Supplementary Material for details*.

*PC, principal component; HBC, hepatitis B core antigen; BMI, body mass index; TV, television; COPD, chronic obstructive pulmonary disease; FEV, forced expiratory volume; FVC, forced volume vital capacity; d, derived variables*.

In contrast, there was no variable contributing more than 57% of variable variance to PC2. The 13 leading contributors were all lung function measures. The PC2 might be interpreted as a component specific to lung function. There was no variable which contributed more than 38% of variances to PC3. Forty-two of the 49 leading variables were the variables of activity monitoring, which were collected in 7 days. For the other PCs, percentages of contributed own variance among leading variables diminished.

## Discussion

We find that it is highly feasible to identify potential stages with representative components derived from population biomarker data based on explicit criteria and statistical tests. The first finding is that the identified PCs seem to represent distinct aspects of human development in a population. The first PC includes a variety of variables that are sensitive to rapid physical development before age 20, such as time spent in physical activities, respiratory symptoms, and the status of drinking and smoking. Two of the leading biomarkers, blood alkaline phosphatase and phosphate levels, are higher among children who are experiencing rapid growth (34). The second and third PCs combine many measures of lung function and physical activities, respectively.

Furthermore, the illustration of the PC trajectories across all age groups provides another important clue for interpretation. The rapid incline of first PC before age 20 shows that a large portion of the variance of the first PC can be attributed to the transition from childhood to adulthood. The trajectories of the second and third PCs resemble the inclines and declines of lung function (1, 35) and physical activities (36, 37), respectively. The sharp changes in PC2 between age 10 and 18 seems to match the rapid expansion of lung capacity. The steady reverse and increasing variation of PC2 after age 20 also correspond to the observed trajectory of lung function, which peaks around age 25 and deteriorate gradually (11). For PC3 that is associated with physical activities, there is sharp change between age 10 and 18. The previous study focusing on this age period confirms the significant decrease in the time spent on physical activities (36). Finally, there are stages of stability and transition identified.

The results are important for several reasons. The first is that this helps to clarify terms that we tend to use without clear definitions or base of evidence. The identified stages can also supplement current understanding of certain periods of life, such as puberty, that is previously defined solely based on sex and physiological development (38). The rapidly changing PC1 before age 20 shows that the development into adulthood involves many more health measures and biomarkers than we had previously assumed. The stable stage of PC1, between age 22 and 33, provides a piece of evidence for the emerging adulthood that some researchers refer to (39, 40).

In addition, the stable stages identified using health-care utilization data help to provide important reflection to these findings (13). The first PC from the Medical Expenditure Panel Survey (MEPS) shows that functional status, employment, and poverty are important determinants (13). There are common stable stages, especially from age 34 to 40, and from 46 to 71. The stage of transition related to rapid physical development observed in the CHMS biomarker data before age 22 seems to correspond to the transition we observed in the MEPS data (13).

The second reason is that identifying the stages is an example of using data to supplement existing knowledge. This is particularly important when we do not have a comprehensive understanding of the relationship between massive biological data and existing information. Our strategy is feasible because there is a large portion of total variance projected to the first PC. We will explore the usefulness of this method in other significant sources of information, such as the Canadian Longitudinal Study of Aging that has recruited and followed up approximately 50,000 subjects for the next two decades (41).

The last reasons is that the stages are important for clinical and epidemiological studies that attempt to group similar individuals of different ages to augment sample sizes in each age category and generate less variable estimates (42, 43). In situations where we do not have a sufficient understanding of how to stratify by age, we suggest formulating similar analysis or adopting potential life stages we have identified using the CHMS data.

### Limitations

There are several limitations to this pioneering study. First, the results of PCA with other biobank data may not be identical to those from the CHMS, especially those collected or created in distinctive manners. Despite the efforts to include expert advice from the National Health and Nutrition Examination Survey (NHANES) and many other biomarker surveys, the CHMS remains a unique initiative to understand health status of Canadians by Statistics Canada (14). We are currently applying this method to other health and aging-related data sets. It requires more work to confirm the generalizability and comparability of the results from the CHMS and many others. The second limitation lies in how the answers to questions in the questionnaire are framed or categorized. The results of PCA can be influenced by how information is gathered, for example categorization of variables and who are the target respondents. The redundant variables or variables documenting similar characteristics can inflate the importance of certain characteristics in PCA (44). Despite efforts to select features with correlation-based methods, we think this method could be further improved.

Third, not all respondents answered all questions or provide samples for all measures because of the eligibility criteria set for certain age groups. For example, only those age 12 years and over were asked about the status of drinking alcohol, and postnatal information, such as days before due day, were collected among those less than 12 years of age (18). This also influences the results of PCA.

Fourth, there may be changes in the questionnaires or alterations in measurement precisions that we could not fully adjust for, despite our exhaustive efforts to review the related documentation. For example, the upper and lower limits of detection for vitamin B12 increased and decreased, respectively, over time (18). Replacing top- or bottom-coding with respective upper or lower detection limits may not be satisfying (45), but this may be more preferable than retrieving original biological samples for second measurements.

Fifth, the cross-sectional design of the CHMS makes it possible to collect data on Canadians of a wide age spectrum, from age 6 to 79. However, we could not infer individual trajectories based on population estimates. The confirmation of the stages of stability and transition still requires the analysis of upcoming data from major cohort studies and trials.

Sixth, the data processing may not be optimal. There remain room for improvement about the methods to impute missing data and recording of the categories. This can be due to the lack of standard or precedent studies. Although the proportions of missing and inapplicability are not high in the variable summary table, we still think there are certain methods that need to be developed and tested for biomarker data.

Seventh, the use of 1-year interval for age groups may not be optimal and should be reviewed. Although our previous research on the use of 2-year intervals did not find clinically significant effects on data analysis based on the use of 2-year intervals (46), we think the effect of 2-year intervals may need to be examined in life stage research.

Finally, this study did not intend nor possess the capacity to identify the external sources that alter the biological measures in populations. For example, the impact of milk fortification (in all cycles) or sunlight exposure (only available in cycle 3) (18) on the blood levels of vitamin D is not assessed.

In summary, the stages and PC values can be influenced by how information is categorized and from whom the information is collected. After acknowledging the limitations of PCA, it is worth paying attention to transitions that are not due to questionnaire changes, for example after age 40, and focusing on the biological fluctuations in this group.

#### Future Research Directions

We are focusing on developing new methods to counteract these limitations. Our first research opportunity is to reassess the underused variables and their relationships to the potential stages across the lifespan. The variables that contribute large portions of variance will be tested first. Second, there are other pieces of information that we would like to link to the first few PCs to determine the usefulness of their values and associations with adverse outcomes, such as frailty and mortality. Third, we will test the representativeness of these potential life stages relative to other single biological measures and socioeconomic status. By beginning with the results of the PCA, we will further test whether there are life style or occupational factors leading to the stability or transitions in life course.

The fourth opportunity is to fine-tune the identified stages. We aim to solve the issues related to changes in questionnaires and the differences in the universes of questions. We will focus on the age groups that are not subject to questionnaire changes or ineligibility for biological measures. The fifth opportunity is to develop the data processing methods that can best reflect the original concepts in the questionnaires or biobank without introducing measurement error to the imputed variables. For example, the processing of the age since disease attack may be improved. The sixth is to adopt other age intervals for the assessment of life stages. There is evidence the age interval may not matter much. However, the effect of other age intervals remains unknown.

Seventh, reproducing the staging research in other major databases will be important. There are other relevant biomarker databases, such as NHANES data in the United States (16). The similarity and differences in the stages identified in the MEPS data (13) will be further studied for the implication on issues such as beginning of aging and opportunities for aging intervention. Finally, we are also interested in understanding the differences between biological (47) and chronological life stages.

## Conclusion

There are stages of stability and transition identifiable from the biomarker data in the CHMS according to the first PCs. The identification of stages between all age groups in the CHMS is the first step to understanding how human biology develops and ages from a population perspective. This staging method is feasible with population biomarker data. The identified stable stages are important for research that relies on a research population with similar characteristics to draw samples for observation or intervention. The stages of transition may be key to understand what contributed to the differences between individuals in later life. In conclusion, the identification of life stages based on biomarker data is the first step to uncover details of potential stages, such as adolescence or aging, and to develop new methods to classify population and biomedical development across life course.

## Declaration

### Required Statement for the Analysis of Statistics Canada Data at the Research Data Centre

The analysis presented in this paper was conducted at the Quebec Interuniversity Centre for Social Statistics, which is part of the Canadian Research Data Centre Network (CRDCN). The services and activities provided by the QICSS are made possible by the financial or in-kind support of the Social Sciences and Humanities Research Council, the Canadian Institutes of Health Research, the Canada Foundation for Innovation, Statistics Canada, the Fonds de recherche du Québec—Société et culture (FRQSC), the Fonds de recherche du Québec—Santé (FRQS) and the Quebec universities. The views expressed in this paper are those of the authors and not necessarily those of the CRDCN or its partners (48).

## Availability of Data and Materials

The CHMS data are available through the RDC program sponsored by Statistics Canada. The data access needs to be approved by Statistics Canada.

## Ethics Statement

This secondary data analysis was approved by the ethics review committee at the Centre Hospitalier de l’Université de Montréal.

## Author Contributions

Y-SC designed and implemented this study. C-JW assisted in drafting the manuscript. H-CW and W-CC reviewed the manuscript.

## Conflict of Interest Statement

The authors declare that the research was conducted in the absence of any commercial or financial relationships that could be construed as a potential conflict of interest.
